# Supplementary material for: Ataxin-7 and Non-stop coordinate SCAR protein levels, subcellular localization, and actin cytoskeleton organization
Source: eLife. 2019 Jul 26;8:e49677. doi: 10.7554/eLife.49677 (PMC6693919; doi:10.7554/eLife.49677)
Supplement: Transparent reporting form [file elife-49677-transrepform.docx]

***eLife’s* transparent reporting form**

We encourage authors to provide detailed information *within their submission* to facilitate the interpretation and replication of experiments. Authors can upload supporting documentation to indicate the use of appropriate reporting guidelines for health-related research (see [EQUATOR Network](http://www.equator-network.org/%20)), life science research (see the [BioSharing Information Resource](https://biosharing.org/)), or the [ARRIVE guidelines](http://www.plosbiology.org/article/info:doi/10.1371/journal.pbio.1000412) for reporting work involving animal research. Where applicable, authors should refer to any relevant reporting standards documents in this form.

If you have any questions, please consult our Journal Policies and/or contact us: [editorial@elifesciences.org](mailto:editorial@elifesciences.org).

**Sample-size estimation**

- You should state whether an appropriate sample size was computed when the study was being designed
- You should state the statistical method of sample size computation and any required assumptions
- If no explicit power analysis was used, you should describe how you decided what sample (replicate) size (number) to use

No power analysis was used. Sample size was determined based on numbers used in similar studies.

Please outline where this information can be found within the submission (e.g., sections or figure legends), or explain why this information doesn’t apply to your submission:

**Replicates**

- You should report how often each experiment was performed
- You should include a definition of biological versus technical replication
- The data obtained should be provided and sufficient information should be provided to indicate the number of independent biological and/or technical replicates
- If you encountered any outliers, you should describe how these were handled
- Criteria for exclusion/inclusion of data should be clearly stated
- High-throughput sequence data should be uploaded before submission, with a private link for reviewers provided (these are available from both GEO and ArrayExpress)

Please outline where this information can be found within the submission (e.g., sections or figure legends), or explain why this information doesn’t apply to your submission:

Figure 1c: mudpit was performed 3 times.

Figure 1d: Western blot was performed 3 times on three biological replicates. A biological replicate is a protein extract taken from cultured S2 cells. The sample size can be found in the figure.

Figure 2a: mudpit was performed time.

Figure 2b and 2c: Reciprocal pulldowns were performed on three biological replicates with a representative immunoblot shown. Biological replicates are protein extracts from separate cell cultures transfected with the stated plasmid or no vector control. Replicates were performed on different days. This information is not included in manuscript.

Figure 2d and E: Immunostaining was performed 2 times and ~ 50 cells were viewed and a representative image is shown. Each replicate represents fixed cells that were transfected on the same day, but immunostained and imaged on separate days. This information is not contained in the manuscript.

Figure 3a: There are three technical replicates for WT and *Atxn7*. Each replicate is a protein extract from larva. The number of replicates is included in the figure.

Figure 3b: There are three biological replicates for *Atxn7* and *Lacz* and two biological replicates for *non-stop* knockdown. Each biological replicate is a protein extract from a different cell culture that had dsRNA added to it. The number of biological replicates is included in the figure.

Figure 3c: Three independent biological replicates were done per condition. A biological replicate is a protein extract from larval brains. The three replicates were dissected and extracted on separate days. The number of biological replicates is included in the figure.

Figure 3d: Three biological replicates were performed and information is found in the figure. Biological replicates are RNA extracted from different cell cultures treated with dsRNA. For each replicate the RQ number is an average two technical replicates; two separate qPCR reactions performed on the same day with the same sample. qPCR data was not included in the final average if it did not meet the standards set for qPCR data: CT above 30 or deltaCT SE of technical replicates >0.25.

Figure 3e: Three biological replicates were performed and this information is found in the figure. Biological replicates are RNA extracted from different larval brains. The RQ number for each replicate is an average of two technical replicates; two separate qPCR reactions performed on the same day with the same sample. qPCR data was not included in the final average if it did not meet the standards set for qPCR data: CT above 30 or deltaCT SE of technical replicates >0.25.

Figure 3e: Five biological replicates were performed and the sample size can be found in the figure. A biological replicate is a protein extract from separate larval brains.

Figure 4a and b: Pulldowns were performed on at least three biological replicates with a representative immunoblot shown. Biological replicates are protein extracts from separate cell cultures transfected with the stated plasmid or no vector control. Replicates were performed on different days. Sample size is included in the figure.

Figure 4c: Three biological replicates were performed. A biological replicate is a protein extract taken from cultured larval brains. The sample size is included in the figure.

Figure 4 d: Three biological replicates were performed. Each biological replicate is a protein extract from a different cell culture that had dsRNA added to it and was transfected. The sample size is included in the figure.

Figure 5a and b: Immunostaining was repeated 2 times and a representative experiment is shown. Each replicate was performed with separate transfections performed on separate days and immunostained and imaged on separate days. For each trial 40-50 cells were measured. The number of individual cells analyzed is shown in the figure. Only numbers that were significantly different in both experiments are shown. Cells were excluded from the analysis if they could not be reliably considered one cell, e.g. two cells on top of each other as determined by the number of DAPI staining bodies or the Phalloidin staining of the membrane.

Figure 6 b: Pulldowns were performed on at three biological replicates with a representative immunoblot shown. Biological replicates are protein extracts from separate cell cultures transfected with the stated plasmid or no vector control. Replicates were performed on different days. Sample size is included in the figure.

Figure 6c: Immunostaining for SCAR was repeated 2 times and data is combined. Each replicate was performed with separate transfections performed on separate days and immunostained and imaged on separate days. 40-50 cells from each condition were measured in each replicate. Cells were only included in the analysis if they were expressing Non-stop at the mid-range. Cells with low non-stop or high non-stop were excluded and this is stated in the manuscript. Cells were excluded from the analysis if they could not be reliably considered one cell, e.g. two cells on top of each other as determined by the number of DAPI staining bodies. The number of individual cells included in the analysis is given in the figure.

Figure 6d: Phalloidin staining was performed one time. Between 20 and 30 cells were analyzed per condition. The number of individual cells analyzed is found in the figure. Cells were excluded from the analysis if they could not be reliably considered one cell, e.g. two cells on top of each other as determined by the membrane visualized with Phalloidin.

Figur 7 a and b: Phalloidin staining was performed on the number of lobes stated in the figure. Each lobe represents a biological replicate. Between 5 and 14 lobes were analyzed per genotype. The number of lobes analyzed is found in the figure. Brains were excluded from the analysis if they were the wrong developmental stage or they could not be reliably measured, e.g. the brain was damaged or improperly mounted as to obscure some of the staining.

Figure 7c: Phalloidin staining was performed on the number of ventral nerve cords stated in the figure. Each nerve cord represents a biological replicate. Between 3 and 7 nerve cords were analyzed per genotype. Brains were excluded from the analysis if they were the wrong developmental stage or they could not be reliably measured, e.g. the brain was damaged or improperly mounted as to obscure some of the staining.

Figure 8: Chaoptin staining was performed on the number of lobes stated in the figure. Each lobe represents a biological replicate. Between 13 and 29 lobes were analyzed per genotype. Brains were excluded from the analysis if they could not be reliably measured, e.g. the brain was damaged or improperly mounted as to obscure some of the staining.

**Statistical reporting**

- Statistical analysis methods should be described and justified
- Raw data should be presented in figures whenever informative to do so (typically when N per group is less than 10)
- For each experiment, you should identify the statistical tests used, exact values of N, definitions of center, methods of multiple test correction, and dispersion and precision measures (e.g., mean, median, SD, SEM, confidence intervals; and, for the major substantive results, a measure of effect size (e.g., Pearson's r, Cohen's d)
- Report exact p-values wherever possible alongside the summary statistics and 95% confidence intervals. These should be reported for all key questions and not only when the p-value is less than 0.05.

Please outline where this information can be found within the submission (e.g., sections or figure legends), or explain why this information doesn’t apply to your submission:

Figure 1d: All data is mean and error is standard error of the mean. This information is in the figure legend.

Figure 3: All data is mean and error is standard error of the mean. This information is in the figure legend.

Figure 4: All data is mean and error is standard error of the mean. This information is in the figure legend.

Figure 5 a and b: Data in bar graph form is mean and error is standard error of the mean. T tests were performed to measure differences between the samples and this information is included in the figure legend. N and exact p-values are presented in the figure.

Figure 5a: In the scatter plot the data shown is the ratio for every cell measured and R squared was determined. This is stated within the manuscript.

Figure 6b: All data is mean and error is standard error of the mean. This information is in the figure legend.

Figure 6c: The data shown is SCAR intensity divided by HA intensity for every cell included in the analysis. The line inside the box is the median. The top of the box is the third quartile and the top of the bar is the maximum. The bottom of the box is first quartile and the bottom of the bar is the minimum. T tests were used to measure significance and this information is included in the figure legend. N and exact p-values are in the figure.

Figure 6d: The data in bar graph form is mean and error is standard error of the mean. T tests were used to measure significance and this is stated in the figure legend. N and exact p-values are presented in the figure.

Figure 7: The data shown in the bar graph is the percentage of brain lobes that had less than 3 defects. A fisher’s exact test was used to determine significance. Exact p-values for significant tests are shown. This is stated within the figure legend. The N is stated in the figure.

(For large datasets, or papers with a very large number of statistical tests, you may upload a single table file with tests, Ns, etc., with reference to sections in the manuscript.)

**Group allocation**

- Indicate how samples were allocated into experimental groups (in the case of clinical studies, please specify allocation to treatment method); if randomization was used, please also state if restricted randomization was applied
- Indicate if masking was used during group allocation, data collection and/or data analysis

This does not apply. Samples were placed into groups based on genetic background.

Please outline where this information can be found within the submission (e.g., sections or figure legends), or explain why this information doesn’t apply to your submission:

**Additional data files (“source data”)**

- We encourage you to upload relevant additional data files, such as numerical data that are represented as a graph in a figure, or as a summary table
- Where provided, these should be in the most useful format, and they can be uploaded as “Source data” files linked to a main figure or table
- Include model definition files including the full list of parameters used
- Include code used for data analysis (e.g., R, MatLab)
- Avoid stating that data files are “available upon request”

Please indicate the figures or tables for which source data files have been provided:
